# Elektrodenplatzierung in der kardialen Devicetherapie

**DOI:** 10.1007/s00399-024-01019-9

**Published:** 2024-05-15

**Authors:** Jonas Wörmann, David Duncker, Till Althoff, Christian Heeger, Roland Tilz, Heidi Estner, Andreas Rillig, Philipp Sommer, Leon Iden, Victoria Johnson, K. R. Julian Chun, Henning Jansen, Tilman Maurer, Sonia Busch, Daniel Steven

**Affiliations:** 1 Abteilung für Elektrophysiologie, Herzzentrum der Uniklinik Köln, Köln, Deutschland; 2 Hannover Herzrhythmus Centrum, Klinik für Kardiologie und Angiologie, Medizinische Hochschule Hannover, Hannover, Deutschland; 3 Klinik für Kardiologie und Angiologie, Charite – Universitätsmedizin Medizin Berlin, Berlin, Deutschland; 4 Department für Rhythmologie, Abteilung für Kardiologie & Internistische Intensivmedizin, Asklepios Klinik Altona, Hamburg, Deutschland; 5 Klinik für Rhythmologie, Universitätsklinikum Schleswig-Holstein (UKSH), Campus Lübeck, Lübeck, Deutschland; 6 Medizinische Klinik und Poliklinik I, LMU Klinikum der Universität München, München, Deutschland; 7 Universitäres Herz- und Gefäßzentrum Hamburg, Universitätsklinikum Eppendorf Hamburg, Hamburg, Deutschland; 8 Med. Klinik für Elektrophysiologie/Rhythmologie, Herz- und Diabeteszentrum NRW , Ruhr-Universität Bochum, Bad Oeynhausen, Deutschland; 9 Klinik für Kardiologie , Herz- und Gefäßzentrum Bad Segeberg, Bad Segeberg, Deutschland; 10 Klinik für Kardiologie und Angiologie, Universitäres Herz- und Gefäßzentrum Frankfurt, Universitätsklinikum Frankfurt, Frankfurt am Main, Deutschland; 11 Cardioangiologisches Centrum Bethanien – CCB, Frankfurt am Main, Deutschland; 12 Elektrophysiologie Bremen, Herzzentrum Bremen am Klinikum Links der Weser, Bremen, Deutschland; 13 Klinik für Innere Medizin II, Asklepios Klinik Nord - Heidberg, Hamburg, Deutschland; 14 Abteilung für Elektrophysiologie, Herz-Zentrum Bodensee, Konstanz, Deutschland; 15 Arrhythmia Section, Cardiovascular Institute (ICCV), CLÍNIC – University Hospital Barcelona, Barcelona, Spanien

**Keywords:** Elektrodenpositionierung, Herzschrittmacher, Implantierbarer Kardioverter-Defibrillator, Septale Stimulation, Apikale Stimulation, Lead positioning, Artificial pacemakers, Implantable cardioverter-defibrillators, Apical stimulation, Septal stimulation

## Abstract

Die Implantation von Elektroden aktiver Herzrhythmusimplantate („cardiac implantable electronic devices“, CIED) erfordert tiefgehendes technisches Verständnis und eine präzise Ausführung. Die Platzierung der Elektroden im rechten Ventrikel und Vorhof hat signifikante Auswirkungen auf die Patientensicherheit und die Effektivität der CIED-Therapie. Insbesondere bei der ventrikulären Platzierung wird dabei der Fokus auf die Unterscheidung zwischen apikaler und septaler Stimulation gelegt. Basierend auf der aktuellen Datenlage, stellt dieser Artikel eine praxisorientierte Anleitung dar, die Implantierende durch die einzelnen Schritte der Elektrodenplatzierung führt. Die Implantation von Elektroden zur physiologischen Stimulation („cardiac resynchronization therapy“, CRT und „conduction system pacing“, CSP) werden an anderer Stelle adressiert und sind nicht Gegenstand dieses Artikels.

## Abstract (en)

The implantation of electrodes for cardiac implantable electronic devices (CIED) requires profound technical understanding and precise execution. The positioning of electrodes in the right ventricle and atrium has significant implications for patient safety and the effectiveness of CIED therapy. Particular focus is given to the distinction between apical and septal stimulation in ventricular positioning. Based on current data, this article provides a practice-oriented guide that leads implanters through the individual steps of electrode positioning. The implantation of electrodes for physiological stimulation (cardiac resynchronization therapy, CRT, and conduction system pacing, CSP) is not addressed in this article.

## Hintergrund

Die Implantation von Herzschrittmachern und implantierbaren Kardioverter-Defibrillatoren (ICD) ist ein etabliertes und häufig durchgeführtes Verfahren in der Kardiologie und Kardiochirurgie. In Deutschland werden pro Jahr knapp 100.000 Erstimplantationen von Schrittmachern und ICD durchgeführt [1, 2]. In vorherigen EP-Basics-Ausgaben wurden bereits venöse Zugangswege, perioperatives Management sowie Elektrodenextraktionen bei aktiven Herzrhythmusimplantaten („cardiac implantable electronic devices“, CIED) adressiert [3–5].

Dieser Artikel gibt einen Überblick zur Durchführung der Platzierung von rechtsventrikulären (RV) und rechtsatrialen (RA) Elektroden sowie Überlegungen zum optimalem Stimulationsort bei *konventionellen* CIED. Die Systeme zur physiologischen Stimulation im Sinne einer kardialen Resynchronisationstherapie (CRT), His- bzw. Linksschenkel-Stimulation sind nicht Gegenstand dieses Artikels und werden separat behandelt.

## Platzierung der rechtsventrikulären Elektrode

### Optimaler Stimulationsort im rechten Ventrikel

Der rechte Ventrikel wird anatomisch in den Einflusstrakt (Dreieck zwischen Trikuspidalklappe, septomarginaler Trabekularisation und Moderatorband), Apex (Moderatorband bis Herzspitze) und Ausflusstrakt (septomarginale Trabekularisation bis Pulmonalklappe) eingeteilt [6]. Traditionell wird der RV-Apex aufgrund der einfachen Erreichbarkeit als bevorzugter Ort für die Platzierung der ventrikulären Elektrode angesehen. Inzwischen wird diskutiert, ob die Platzierung am RV-Septum gegenüber der apikalen Lage überlegen sein könnte [7].

Die Unterscheidung zwischen apikal und septal erfolgt anhand von anatomischen sowie prozeduralen Gesichtspunkten. Da die Begrifflichkeiten septal und apikal teilweise heterogen verwendet werden, legt dieser Artikel folgende Definitionen zugrunde:

1. Apikale Platzierung: Platzierung der Elektrode in der Spitze (dem Apex) des rechten Ventrikels. Eine apikoseptale Platzierung beschreibt dabei eine Platzierung im Apex, bei der die Elektrode in Richtung Septum und nicht in Richtung der freien Wand zeigt.
2. Septale Platzierung: Platzierung entlang des interventrikulären Septums (hoch- oder mittseptal).

In den Leitlinien der ESC von 2021 werden sowohl die apikale als auch die septale Position unter dem Verweis auf die heterogene Studienlage gleichwertig empfohlen [8]. Laut einem EHRA-Survey von 2013 verwendete rund die Hälfte der 62 befragten implantierenden europäischen Zentren den RV-Apex als präferierten Stimulationsort (47 %; [9]). Auch eine Umfrage unter den Autoren dieses Artikels aus 12 Zentren ergab, dass ein Drittel der Zentren primär septale Platzierungen durchführen, während 50 % apikoseptale und 17 % apikale Platzierungen präferieren. Der Gesamtanteil an hoch- bis mittseptal implantierten Elektroden wurde in 60 % der Zentren auf < 30 % geschätzt. Die apikale bzw. apikoseptale Platzierung entspricht demnach auch weiterhin in einem Großteil der Zentren der klinischen Realität.

Auf elektrische Parameter wie Reizschwelle, Sensing und Impedanz scheint die Elektrodenposition keinen direkten Einfluss zu haben [10]. Jedoch besteht bezüglich der Komplikationsrate ein Nachteil der apikalen Platzierung durch ein über 3‑faches Risiko einer Perforation [11].

Zusätzlich gibt es Hinweise, dass eine Platzierung im RV-Apex im Vergleich zum RV-Septum aufgrund der asynchronen Stimulation des linken Ventrikels (LV) mit zunehmendem Stimulationsanteil vermehrt zu einer sog. schrittmacherinduzierten Kardiomyopathie mit Verschlechterung der LV-Funktion, Herzinsuffizienzsymptomen und erhöhter Mortalität führen kann [12, 13].

Dies lässt sich durch die unphysiologische Stimulation der apikalen Position im Vergleich zur septalen erklären: Durch die frühe Erregung des RV-Apex und die spätere Erregung des RV-Septums und damit des LV erfolgt die stimulierte Erregung teilweise entgegengesetzt der physiologischen Erregungsausbreitung. Dies hat einen atypischen iatrogenen Linksschenkelblock (LSB) zur Folge, der zu interventrikulärer Dyssynchronie und in der Folge zur Reduktion der LV-Funktion führen kann.

In Untersuchungen, in denen mittels 3D-Mapping-Verfahren an verschiedenen Positionen im RV stimuliert wurde, konnte gezeigt werden, dass die beste Kontraktilität echokardiographisch durch mitt- bis hochseptale Stimulation erreicht wird [14]. Dies drückt sich auch darin aus, dass durch septale Stimulation schmalere QRS-Komplexe erreicht wurden als an anderen Positionen [15].

Zuletzt ist auch die Möglichkeit einer iatrogenen Trikuspidalklappeninsuffizienz bzw. die mögliche Verschlechterung einer vorbestehenden Trikuspidalklappeninsuffizienz durch die Passage der Klappe und abhängig von der anschließenden Lage der Elektrode stärker in den Fokus gerückt [16]. In einer randomisierten Studie mit 128 Patienten hatte die Elektrodenposition keinen Einfluss auf die Häufigkeit einer Trikuspidalklappeninsuffizienz [17]. Auf der anderen Seite konnte in einer retrospektiven Arbeit mit 396 Patienten gezeigt werden, dass eine apikale Position der RV-Elektrode durch ein Impingement des posterioren Segels eher zu einer relevanten Trikuspidalklappeninsuffizienz führt als eine septale, bei welcher der Durchtritt durch die Mitte der Trikuspidalklappe erfolgt. Durch den posterioren Durchtritt trat eine schwere Insuffizienz bei 57,6 % vs. 9,7 % bei mittigem Durchtritt auf [18].

Während die ESC-Leitlinie von 2021 beide Positionen gleichwertig benennt, befürwortet das Consensus Statement der EHRA aus dem gleichen Jahr, die septale Platzierung der apikalen vorzuziehen, wobei bezüglich der Platzierung keine eindeutige Empfehlung erfolgt [8, 10].

Die Autoren dieses Artikels schließen sich dem EHRA Consensus Statement an, präferenziell eine septale Platzierung durchzuführen, um unerwünschte Komplikationen sowie das Risiko für schrittmacherinduzierte Kardiomyopathien zu minimieren. Dies gilt im Besonderen, wenn ein hoher Stimulationsanteil zu erwarten ist und bei Patienten mit bereits eingeschränkter LV-Funktion. Wenn eine apikale Platzierung gewählt wird, sollte eine Platzierung an der freien Wand vermieden werden (apikoseptale Platzierung).

Die rechtsventrikuläre Elektrode benötigt ausreichend, aber auch nicht zu viel Spiel (*Schlaufe*), was ggf. durch Fluoroskopie in tiefer Inspiration kontrolliert werden sollte. Bei zu kurzem Spiel wird das septale Trikuspidalklappensegel gegen die septale Wand („ventriculoinfundibular fold“) gedrückt, bei zu viel Spiel wird das posteriore Trikuspidalklappensegel gegen den die freie basale Wand des rechten Ventrikels gedrückt. In beiden Fällen kann eine höchstgradige („torrential“) Trikuspidalklappen-Insuffizienz resultieren.

### ICD-Elektrodenplatzierung und „Sonderfälle“

Ein besonders relevantes Thema ist die Platzierung von ICD-Elektroden, analog zu Schrittmacherelektroden speziell der Vergleich zwischen RV-Septum und RV-Apex. Traditionell wird auch hier der RV-Apex aufgrund seiner guten Erreichbarkeit und der einfach durchzuführenden Elektrodenimplantation favorisiert. Das RV-Septum stellt jedoch auch bei ICD eine Lokalisation dar, die insbesondere bei Patienten mit zusätzlichem Stimulationsbedarf die zuvor erläuterten Vorzüge bieten kann. Außerdem wurden auch bei ICD-Elektroden bei der Platzierung am RV-Septum geringere Komplikationsraten als bei Platzierung am RV-Apex gezeigt [11].

Hinsichtlich der Defibrillationsschwelle und möglicher ineffektiver Schocks konnte eine Subanalyse der SIMPLE-Studie mit 2475 Patienten keine Unterschiede zwischen apikaler und nichtapikaler Lage identifizieren [19]. Ebenso zeigte die randomisiert-prospektive SEPTAL-Studie mit 215 Patienten, dass eine septale Platzierung der apikalen hinsichtlich Durchführbarkeit und Effektivität der Schockabgaben nicht unterlegen ist [20].

In Zusammenschau der vorliegenden Daten sollte nach Meinung der Autoren daher auch für ICD-Elektroden die septale gegenüber der apikalen Position vorgezogen werden.

Für CRT-Patienten ergab eine retrospektive Analyse mit 313 Patienten, dass eine septale Platzierung sich positiv auf die Resynchronisation auswirkt und schmalere QRS-Komplexe erreicht werden als bei apikaler Platzierung [21]. Dies lässt sich dadurch erklären, dass der zeitliche Unterschied der Aktivierung zwischen RV- und LV-Elektrode für die erfolgreiche Resynchronisationstherapie entscheidender ist als der anatomische Abstand. Der zeitliche Abstand ist durch die relativ zum lateralen LV frühere Aktivierung des Septums größer als bei der apikalen [22]. Es werden weitere randomisierte Studien benötigt, um die optimale Position für dieses Patientenkollektiv zu bestimmen.

Einen weiteren Sonderfall stellen Patienten mit hypertroph obstruktiver Kardiomyopathie (HOCM) dar, bei denen eine AV-sequenzielle Stimulation mit verzögerter Aktivierung des Septums erwünscht ist, da sie zu einem Abfall des Gradienten über dem linksventrikulären Ausflusstrakt (LVOT) führen kann [23].

Dies wird durch die verspätete systolische Aktivierung und damit Kontraktion des hypertrophen Septums erreicht. Die Septumwulst obstruiert erst zum Ende der Systole den LVOT, was zu einem verbesserten Auswurf in der frühen und mittleren Systole führt.

Aktuell besteht bei diesen Patienten eine Klasse-IIb-Indikation zur Schrittmacherimplantation als Alternative zur Myektomie oder der transarteriellen Ablation der Septumhypertrophie (TASH; [8, 24]). Es konnte gezeigt werden, dass die apikale Stimulation hinsichtlich der Reduktion des LVOT-Gradienten und der Symptomatik gegenüber der septalen Stimulation überlegen ist [25]. Die Gruppe der HOCM-Patienten bildet also ausdrücklich eine Ausnahme von der ansonsten geltenden Empfehlung zur septalen Platzierung, die mit den Besonderheiten der Pathophysiologie der Myokarderkrankung zusammenhängt.

## Technische Durchführung der Elektrodenplatzierung

### Rechtsventrikuläre Elektrode

Im Folgenden wird die Platzierung der RV-Elektrode beschrieben. Dabei wird sich der Artikel auf die aktiv mittels Schraube fixierbaren Modelle beschränken, da passive Elektroden (z. B. Ankerelektroden) in der klinischen Routine praktisch keine Rolle mehr spielen.

Nach Etablierung des venösen Zugangswegs erfolgt das Einbringen der Elektroden [4]. In aller Regel erfolgt bei allen Systemen dabei zunächst die Platzierung der RV-Elektrode, um eine sichere rechtsventrikuläre Stimulation zu etablieren und eine Dislokation weiterer Elektroden zu vermeiden.

Jede Elektrode ist vor Einbringen auf Unversehrtheit zu überprüfen. Außerdem sollte geprüft werden, ob die Schraube gänzlich in der Elektrodenspitze verschwindet. Bei der Handhabung des Stylets sollte darauf geachtet werden, dass die Handschuhe frei von Blut sind, da es sonst zu Verklebungen im Elektrodenlumen kommen kann.

**Abb. 1:**
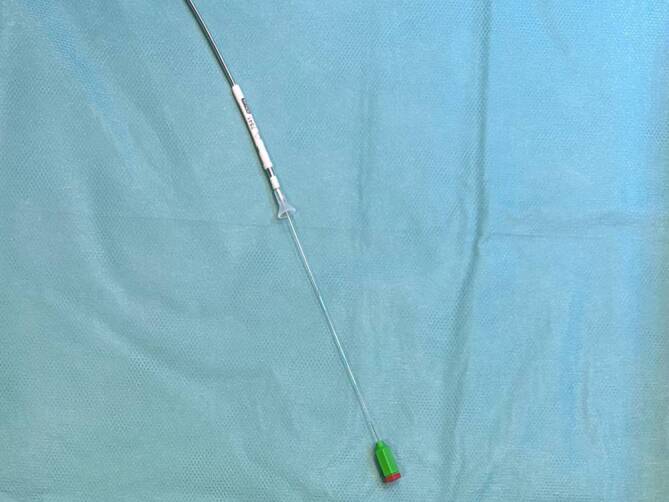
Elektrode (Ingevity™, Boston Scientific, Marlborough, MA, USA) mit 2–3 cm zurückgezogenem Stylet

Unabhängig vom Stimulationsort ist die Passage der Trikuspidalklappe aus dem rechten Vorhof der erste Schritt zur RV-Elektroden-Platzierung und stellt gerade bei den ersten Implantationen eine Herausforderung dar. Hierfür wird in der Regel das interne Stylet vorgebogen und die Elektrode mit vorgeschobenem Stylet in posteroanteriorer (p.-a.) oder RAO-Projektion über die Trikuspidalklappe geführt. Es empfiehlt sich, dabei das Stylet ca. 2–3 cm zurückgezogen zu lassen (Abb. 1). So bleibt die Spitze der Elektrode atraumatisch und kann zur Sondierung der Trikuspidalklappen-Ebene genutzt werden.

**Abb. 2:**
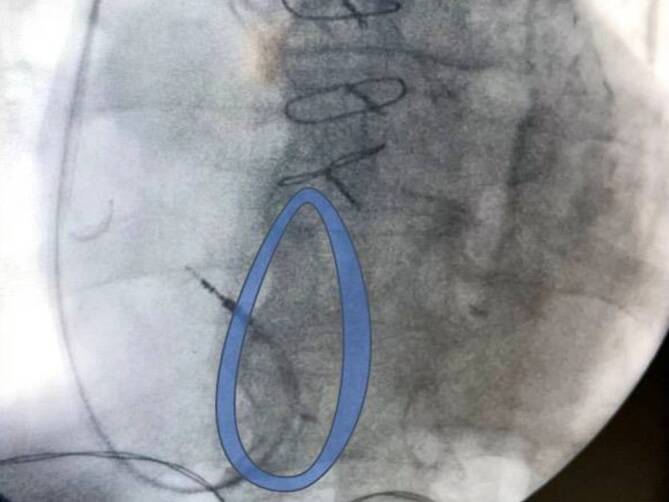
Intraoperative fluoroskopische posteroanteriorer (p.-a.)-Projektion mit Durchtritt der rechtsventrikulären (RV)-Elektrode über die Trikuspidalklappe (*blau*) mittels vorgebogenem Stylet. Die Elektrodenspitze bleibt dabei atraumatisch durch einen Rückzug des Stylets um 2–3 cm. Der Durchtritt erfolgt mit der atraumatischen Elektrodenschlaufe

Alternativ kann die Elektrodenspitze mit weiter zurückgezogenem Stylet an der rechtsatrialen Wand angestellt und dann vorgeschoben werden. So bildet der proximale Teil der Elektrode eine Schlaufe und prolabiert in den RV. Dann kann das gerade Stylet vorgeschoben werden, um die Elektrodenspitze in den RV zu einzubringen (Abb. 2). Durch die Elektrode mechanisch induzierte ventrikuläre Extrasystolen (VES) bzw. nichtanhaltende ventrikuläre Tachykardien (nsVT) sind hinweisend auf eine Position im RV. Ein Ausbleiben von VES oder nsVT können auf eine Fehllage im Sinus coronarius (CS) hinweisen. Ein weiterer Hinweis auf eine Fehllage kann sich aus den abgeleiteten intrakardialen Elektrogrammen (Vorhofsignal und Ventrikel-Fernfeld im CS) und aus der fluoroskopischen Darstellung in weiteren Projektionen (siehe Absatz „Platzierung im RV-Apex“ und „Platzierung am RV-Septum“) ergeben.

### Platzierung im RV-Apex

Bei einer apikalen Platzierung sollte nach Passage der Trikuspidalklappe die Elektrode zunächst in den rechtsventrikulären Ausflusstrakt (RVOT) bzw. in die Pulmonalarterie platziert werden. Dies gelingt mit dem vorgebogenen Stylet oder ebenfalls durch das Anstellen (mit zurückgezogenem Stylet) und konsekutiv Prolabieren der Elektrode in den RVOT. Vom RVOT aus kann dann auf ein gerades Stylet gewechselt und die Elektrode leicht zurückgezogen werden, bis sie in den RV *fällt*. Mit leicht zurückgezogenem Stylet kann sie dann weiter in den RV-Apex vorgebracht und Richtung Septum gedreht werden, um das Risiko einer Perforation zu minimieren. Bei Fixierung der Elektrode mit dann vollständig vorgebrachtem Stylet sollte die Spitze leicht nach inferior zeigen.

**Abb. 3:**
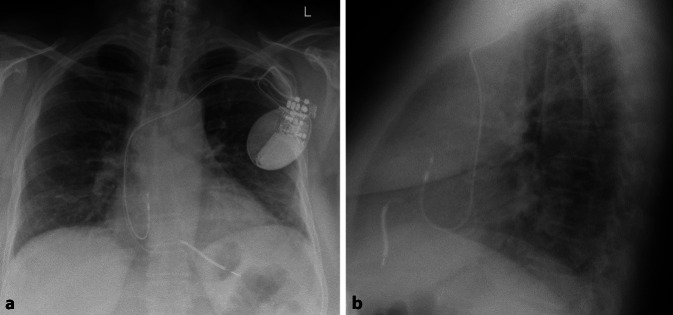
Röntgen-Thorax bei einer Patientin mit apikaler ICD-Elektrode bei Einkammer-ICD. **a** p.-a.-Projektion. **b** Laterale Projektion

**Abb. 4:**
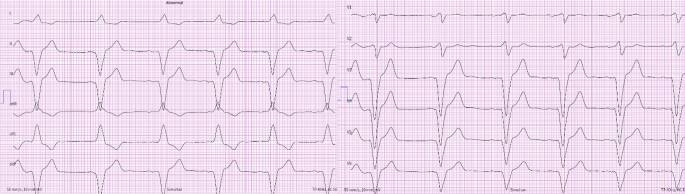
12-Kanal-EKG (50 mm/s): Ventrikuläre Stimulation im VAT-Modus bei apikaler RV-Stimulation. Erkennbar an der superioren Achse (II, III, aVF neg. und I, aVL pos.) und der negativen Konkordanz über der Brustwand, sowie breitem QRS-Komplex (170 ms)

Die RAO 30°-Projektion kann hilfreich sein, sowohl den Eintritt über die Trikuspidalklappe als auch das Vorbringen in den RV-Apex zu beurteilen, da sich der RV nur in dieser Angulation in seiner gesamten Länge projiziert. Eine potenzielle Fehllage im CS kann am besten in LAO 40–60° beurteilt werden [7]. Unter leichtem Anpressdruck mit Aufstellen der Elektrode kann daraufhin das Einschrauben erfolgen. Die sichere Fixierung der Elektrode wird anschließend mit einer schnellen Rückziehbewegung des Stylets und gleichzeitigem Vorbringen der Elektrode überprüft. Vor der Annaht sollte auf genügend Spiel der Elektrode im distalen Drittel geachtet werden, so dass diese die Form eines Schuhabsatzes bildet (Abb. 3). Dabei ist zu bedenken, dass im Stehen oder bei tiefer Inspiration das Spiel der Elektrode im Vergleich zum intraoperativen Bild häufig abnimmt bzw. erhöhter Zug auf der Elektrode ist [7]. Im EKG zeigt sich unter apikaler Stimulation eine superiore Achse mit Breitem QRS-Komplex und negativer Konkordanz über der Vorderwand (Abb. 4)

### Platzierung am RV-Septum

**Abb. 5:**
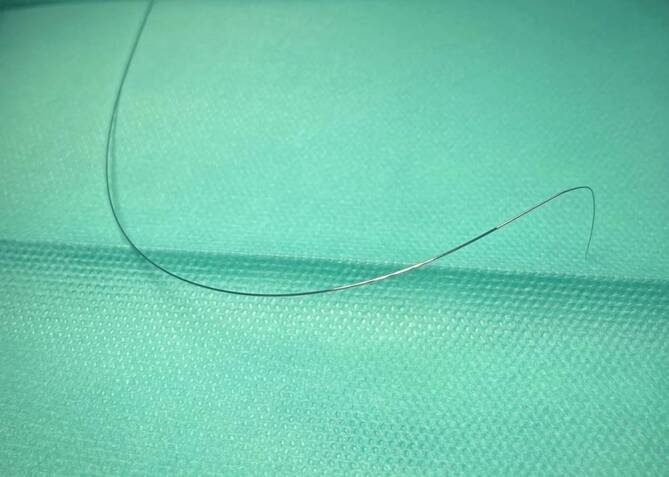
Vorgebogenes Stylet mit großer proximaler und scharfer kurzer distaler Biegung nach septal. (Mod. nach [28])

Zur septalen Platzierung der ventrikulären Elektrode wird zunächst analog zur apikalen Platzierung die Elektrode in den RVOT vorgebracht (s. oben). Daraufhin wird auf ein dreidimensional vorgebogenes Stylet gewechselt. Die Vorbiegung des Stylets kann dabei mit dem Daumen oder alternativ mit einer 10- und einer 5‑ml-Spritze erfolgen. Es erfolgt eine proximale weite (mit der 10-ml-Spritze) und daraufhin (mit der 5‑ml-Spritze) eine scharfe distale nach septal (posterior) zeigende Vorbiegung ca. 2 cm proximal der Spitze in Form eines *Schwanenhalses*. Der Radius des Stylets sollte dabei jeweils an die Größe des Vorhofs und Ventrikels angepasst werden (Abb. 5; [7, 26, 27]). Es ist hilfreich, das Stylet zuvor mit NaCl anzufeuchten.

Diese dreidimensionale Vorbiegung wurde von Mond et al. erstbeschrieben und steht auch als Mond-Stylet™ (Abbott Laboratories, Chicago, IL, USA) kommerziell zur Verfügung [27, 28]. Es existieren außerdem Variationen von Burri (mit größerer proximaler Kurve) und Srivatasa (kleinere proximale und größere distale Kurve) sowie Osmanick (größere proximale und distale Kurve; [29]).

Unter fluoroskopischer Kontrolle erfolgt dann der Rückzug der Elektrode aus dem RVOT, die dabei vorsichtig nach septal (gegen den Uhrzeigersinn) gedreht wird, bis sie an das hohe bzw. mittlere Septum *fällt*.

**Abb. 6:**
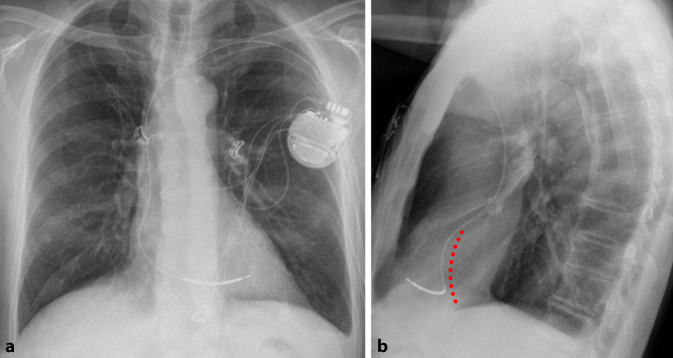
Röntgen-Thorax nach Implantation eines Einkammer-ICD’s. **a** In der p.-a.-Projektion scheint eine mittseptale Position vorzuliegen. **b** In der lateralen Ansicht zeigt sich jedoch eine deutlich nach anterior zur freien RV-Wand zeigende Elektrodenspitze (*rot* Septum)

Eine mögliche Schwierigkeit der septalen Platzierung ist eine Fehllage im anterioren RV bzw. an der freien Wand: In der p.-a.- bzw. LAO-Projektion (40–60°) kann fälschlicherweise eine septale Position angenommen werden, obwohl sich die Elektrode tatsächlich im anterioren oder anteroseptalen RV befindet (Abb. 6; [26, 30]). In einer randomisierten Studie mit 59 Patienten zeigte sich nach echokardiographischer Kontrolle in lediglich 54 % eine korrekte septale Lage [31]. Eine weitere Arbeit, in der die angegebene fluoroskopische Lage mit postoperativen CT-Bildern verglichen wurde, kam auf nur 21 % erfolgreich septal implantierte Elektroden [32].

Durch diese anteriore *Fehllage* wird eine Zunahme von schrittmacherinduzierter Kardiomyopathie sowie von Perforationen beschrieben. Außerdem gibt es einzelne Fallberichte über akute Myokardinfarkte durch die räumliche Nähe zur LAD [33–36]. Die anteriore Lage sollte daher möglichst vermieden werden.

**Abb. 7:**
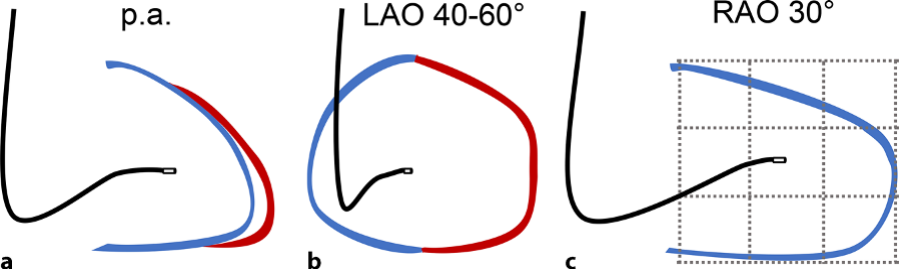
Schematische Darstellung einer mittseptalen Lage der RV-Elektrode. In allen 3 Projektionen zeigt die Elektrode Richtung 3 Uhr. *Blau* rechter Ventrikel (RV) und Trikuspidalklappe (*hellblau*), *rot* linker Ventrikel (LV) und Mitralklappe (*hellrot*). **a** p.-a.-Projektion. **b** In LAO 40–60° kann eine Lage an der freien RV-Wand (Elektrode zeigt nach 7–11 Uhr), eine apikale Lage (Elektrode zeigt nach 5–6 Uhr) oder Fehllage im Sinus Coronarius (CS) (Elektrode überschreitet die Bildmitte) ausgeschlossen werden. **c** In RAO 30° ist das Zielgebiet das mittlere Feld des in 3 × 3 Felder aufgeteilten RV

Um dies zu erreichen, kann die fluoroskopische Lagekontrolle, neben einer p.-a.- und LAO-Projektion (40–60°), um die RAO-30°-Projektion ergänzt werden [6, 7]. In allen 3 Projektionen sollte die Elektrodenspitze Richtung 3 Uhr zeigen. In der RAO-Projektion wird der Herzschatten des RV in 3‑mal 3 Felder unterteilt, wobei die Zielregion für die mittseptale Lage das zentrale Feld bildet (vereinfacht: die Mitte des RV-Herzschattens in RAO). Dies ist schematisch in Abb. 7 dargestellt. Mit diesem Vorgehen in Kombination mit dreidimensionalem Stylet kann eine Erfolgsrate von bis zu 95 % erreicht werden [26, 30].

Alternativ werden verschiedene Verfahren diskutiert, eine individualisierte LAO-Projektion, angepasst auf die Herzachse, zu etablieren. Diese sind jedoch in der klinischen Anwendung weniger etabliert und werden daher an dieser Stelle nicht detailliert behandelt [37].

Nach der Lagekontrolle wird die Elektrode vorsichtig gegen das Septum vorgeschoben (evtl. mit geringem Druck gegen den Uhrzeigersinn bzw. nach posterior) und eingeschraubt.

Die abschließende Lagekontrolle nach Fixierung erfolgt durch ruckartiges Herausziehen des Stylets und gleichzeitiger Vorwärtsbewegung der Elektrode. Bei unzureichender Fixierung fällt die Elektrode dabei in den RV.

Als weiterer Hinweis auf die korrekte mittseptale Lage kann ein schmaler stimulierter QRS-Komplex mit vergleichsweise früher R/S-Transition in den Brustwandableitungen hinzugezogen werden. Dies ist im operativen Setting mit häufig nur einer bis 3 Monitorableitungen jedoch nicht immer umsetzbar.

Ebenso wie bei der apikalen Position sollte auf ausreichend Spiel der Elektrode geachtet werden. Bei einer zu straff gelegten septalen Elektrode, ebenso aber bei zu großzügigem Spiel droht eine Dislokation durch die vermehrte Bewegung der Elektrodenspitze. Insgesamt ergeben sich jedoch für die septale Platzierung bei korrekter Anwendung keine Hinweise auf höhere Dislokationsraten in der Akutphase oder im Follow-up im Vergleich zur apikalen Lage [10].

Abschließend sollte eine erneute Lagekontrolle in p. a. und zumindest LAO 40–60° erfolgen (optimal zusätzlich in RAO 30°).

**Abb. 8:**
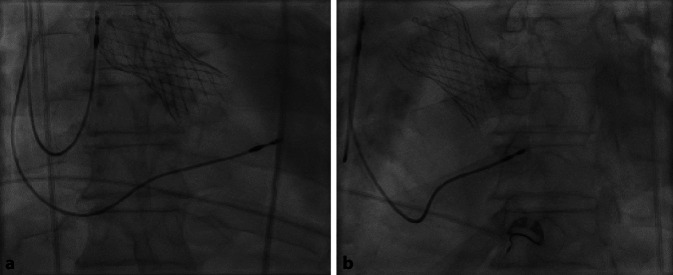
Rechtsatriale (RA)- und septale RV-Elektrode in p.-a. bei einem Patienten nach transfemoraler TAVI und postprozeduralem AV-Block III°. **a** In der p.-a.-Projektion ist die septale Lage nur eingeschränkt zu beurteilen. **b** In der LAO 40°-Projektion zeigt sich eine nach septal (*3* *Uhr*) zeigende Elektrodenspitze

Die intraoperativen fluoroskopischen Lagekontrollen, der postoperative Röntgen-Thorax sowie das schrittmacherstimulierte EKG bei septaler Lage der RV-Elektrode werden in Abb. 8, 9 und 10 gezeigt.

**Abb. 9:**
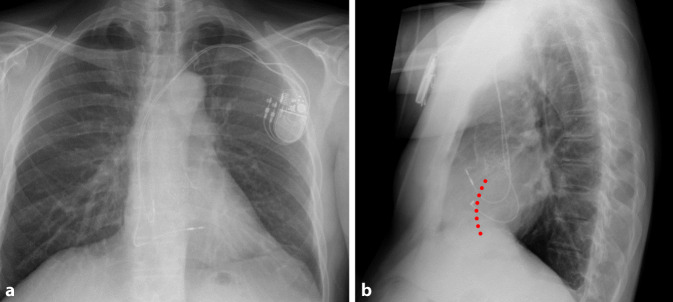
Postoperativer Röntgen-Thorax bei o. g. Patienten. **a** p.-a.-Projektion. **b** In der lateralen Ansicht zeigt sich eine nach dorsal Richtung Septum (*rot*) orientierte Elektrodenspitze

**Abb. 10:**
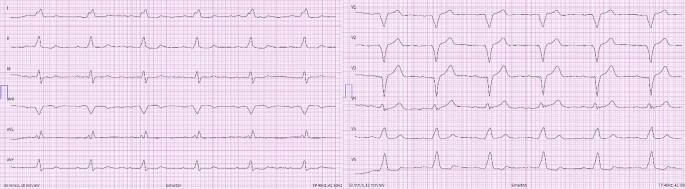
12-Kanal-EKG (50 mm/s): Ventrikuläre Stimulation im VAT-Modus des o. g. Patienten. Zu beachten ist der (bei LSB-Konfiguration) schmale QRS-Komplex (135 ms), der Linkslagetyp und die frühe R/S-Transition als Ausdruck der frühen Aktivierung des LV

Unabhängig vom Stimulationsort kann vor Fixierung der Elektrode eine Bestimmung des Sensings (Amplitude des ventrikulären Signals) erfolgen, um nicht unnötig viele Fixierungsversuche mit potenzieller Perforationsgefahr zu benötigen. Bei der aktiven Fixierung ist nicht die Anzahl der Umdrehungen, sondern die fluoroskopische Sichtbarkeit der ausgeschraubten Helix bzw. je nach Hersteller der Marker entscheidend [7].

Nach dem Einschrauben der Elektrode müssen Sensing, Reizschwelle und Impedanz bestimmt werden. Die jeweiligen von der EHRA empfohlenen Grenzwerte sind in Tab. 1 abgebildet und sind als Minimalwerte zu verstehen [7].

**Tab. 1:** Grenzwerte für die rechtsatriale (RA-) und rechtsventrikuläre (RV-) Elektrode nach Empfehlung des EHRA Consensus Statements von 2021

	RA	RV
Sensing	≥ 1,5 mV	≥ 4 mV
Reizschwelle (bei 0,5 ms)	≤ 1,5 V	≤ 1,5 V
Impedanz*	400–1200 Ω	400–1200 Ω

* Kann herstellerabhängig abweichen

**Abb. 11:**
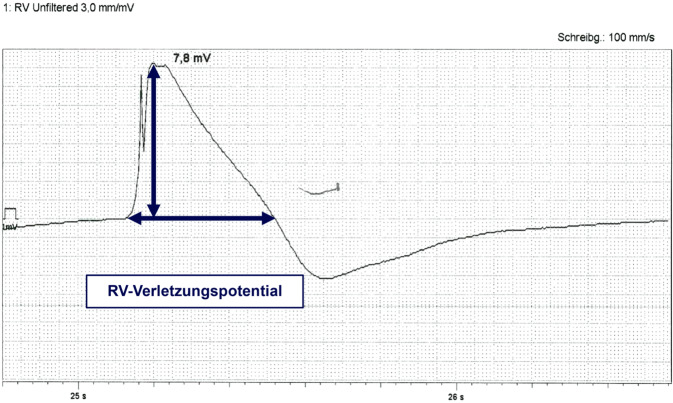
Intrakardiales Elektrogramm nach dem Einschrauben in das rechtsventrikuläre (RV-) Myokard mit deutlicher Verlängerung des Signals und ST-Hebung

Zusätzlich sollte das abgeleitete intrakardiale Signal in Hinblick auf das sog. Verletzungspotenzial beurteilt werden, das durch das Einschrauben in das Myokard entsteht. Es zeichnet sich durch eine längere Dauer und eine ST-Hebung des intrakardialen Signals aus und kann auf dem Programmiergerät evaluiert werden. Das Vorliegen eines hohen Verletzungspotenzials (> 50 ms Verlängerung des Potenzials und > 5 mV ST-Hebung) spricht für einen guten Elektroden-Myokard-Kontakt und somit eine gute Fixierung. Ein negativer/diskordanter Verletzungsstrom weist auf eine Perforation hin und sollte eine Neupositionierung der Elektrode nach sich ziehen [38]. Die Reizschwelle fällt in der Regel bei hohem Verletzungspotenzial weiter ab, während das Sensing sich sowohl vergrößern als auch verkleinern kann. Daher lohnt es sich, bei hohem Verletzungspotenzial auch bei primär suboptimalen Messwerten wenige Minuten zu warten und erneut zu messen, bevor eine Neuplatzierung vorgenommen wird. Ein typisches Verletzungspotenzial ist in Abb. 11 dargestellt.

## Platzierung im rechten Vorhof

Die häufigsten Komplikationen der RA-Elektroden-Implantation betreffen Dislokationen sowie Perforationen [39]. Um diese zu vermeiden, empfiehlt sich ein strukturiertes Vorgehen bei der Platzierung.

Die traditionelle Position der RA-Elektrode ist das rechte Vorhofohr (RAA). Es wird diskutiert, ob eine Stimulation des Bachmann-Bündels oder des interatrialen Septums das Auftreten von Vorhofflimmern senkt. Hierzu liegt jedoch keine eindeutige Evidenz vor [40]. Ebenso zeigten sich keine Vorteile hinsichtlich der Komplikationsrate, weshalb weiterhin die RAA-Position als Standard empfohlen wird [7].

Um die Vorhofelektrode im RAA zu platzieren, wird die Elektrode zunächst mit geradem Stylet über die V. cava superior in den rechten Vorhof eingebracht. Daraufhin wird das Stylet gegen ein J‑förmiges gewechselt. Unter vorsichtigem Ziehen an der Elektrode rutscht diese mit der aufgestellten Spitze in das RAA.

Dabei ist darauf zu achten, dass die Elektrode in p.-a. in Richtung des Untersuchers zeigt. Die Position der Elektrodenspitze lässt sich dabei durch Drehen des Stylet steuern. Eine Drehung im Uhrzeigersinn bewirkt eine laterale, eine gegen den Uhrzeigersinn eine mediale Bewegung.

Unter weiterem Zug wird die Elektrode leicht angestellt, so dass sich ein Winkel von knapp unter 90° ergibt. Die korrekte Lage im RAA lässt sich (bei Patienten im Sinusrhythmus) durch die typische *Scheibenwischer*-Bewegung überprüfen. Eine Überprüfung der Lage in LAO und/oder RAO-Projektionen kann sinnvoll sein, um z. B. eine Fehllage an der RA-Hinterwand oder im lateralen RA auszuschließen. Liegt die Elektrode regelrecht im RAA, zeigt sie nach anterior (Spitze zeigt in RAO Richtung 2 Uhr, in LAO Richtung 10–11 Uhr; [7, 41]).

Eine laterale Lage im RAA sollte vermieden werden, da die Wanddicke im lateralen RAA reduziert ist [42].

Ebenso sollte eine zu mediale Position wegen der Nähe zur Aorta und potenziell lebensbedrohlichen Komplikationen bis zur Aortenperforation vermieden werden [43].

Daraufhin erfolgt die Fixierung mit angestellter Elektrode. Durch vorsichtiges Vorschieben der Elektrode und Zug des Stylets wird die Elektrode in ihre typische Position mit J‑förmigem Spiel gebracht. Um das endgültige Spiel zu beurteilen, ist es sinnvoll, das Stylet soweit zurückzuziehen, dass die Biegung im Venenwinkel zu liegen kommt.

Bei der Platzierung der RA-Elektrode ist zu beachten, dass die Messwerte für Sensing und Reizschwelle teilweise erheblich durch Zu- und Abnahme des Spiels schwanken. Dies lässt sich durch den veränderten Anstellwinkel und somit Vektor des Dipols der Elektrodenspitze erklären. Es empfiehlt sich daher, vor einer Umplatzierung aufgrund schlechter Werte eine Messung mit mehr oder weniger Spiel durchzuführen.

Zur Beurteilung der Stabilität der RA-Elektrode, die häufiger disloziert als die RV-Elektrode, ist das oben erwähnte ruckartige Zurückziehen des J‑Stylets geeignet [7, 44]. Eine weitere Möglichkeit ist das Vorbringen eines geraden Stylets in die J‑förmig liegende Elektrode zur zusätzlichen Überprüfung der Fixierung.

Eine Positionierung am septalen Vorhofdach (*Bachmann-Bündel*) erfolgt zunächst ähnlich dem rechten Vorhofohr, es können die konventionellen J‑förmigen Mandrins benutzt werden. Die Elektrode wird in p.-a.-Projektion in den hohen Vorhof vorgeschoben, wo die Spitze nach medial (nicht lateral) zeigen muss. Dann wird die Projektion auf LAO 40° gedreht. Bei Lage im RAA zeigt die Elektrodenspitze in Richtung ca. 9–10 Uhr, bei Lage am septalen Vorhofdach auf ca. 2–3 Uhr. Die Elektrode kann aus dem RAA durch eine Drehung gegen den (1. Versuch) oder mit dem (2. Versuch) Uhrzeigersinn um 180° an das Bachmann-Bündel gedreht werden. Meist gelingt dies einfacher, wenn die Elektrode bei dieser Drehbewegung etwas vorgeschoben wird. Wenn die Elektrode am septalen Vorhofdach zu liegen kommt, wird dies beim vorsichtigen Zurückziehen dadurch bestätigt, dass die Spitze an gleicher Stelle liegen bleibt und nur die Schlaufe flacher wird.

## Mögliche Komplikationen bei der Elektrodenplatzierung

Eine ausführliche Auflistung der perioperativen Komplikationen ist in der EP-Basics-Ausgabe zum perioperativen Management (Krieger et al. 2024) verfügbar [3].

Die häufigsten Komplikationen im Rahmen der eigentlichen Platzierung sind Dislokationen. Diese betreffen die RA-Elektrode, wie oben genannt 1,5- bis 2‑mal häufiger als die RV-Elektrode [44]. Die zweithäufigste Komplikation ist die Perforation bzw. Tamponade, gefolgt von selteneren Komplikationen [7, 44].

Durch ein strukturiertes Vorgehen und die korrekte Anwendung der oben beschriebenen Techniken können viele dieser teils lebensbedrohlichen Komplikationen vermieden werden.

Die häufigste Elektrodenkomplikation, die Dislokation, ist bei adäquater Implantationstechnik für aktiv fixierbare Elektroden (*Schraubelektroden*) signifikant seltener als für passiv fixierbare Elektroden (*Ankerelektroden*). Hierzu muss jedoch die Röntgenkennung, an der eine komplette Extension der Helix erkennbar ist, bekannt sein. Während bei mehreren Elektrodentypen die Schraube bei kompletter Extension 2 Umdrehungen distal eines Röntgenmarkers sichtbar wird, ist bei anderen Elektroden die Schraube immer distal eines Markers zu sehen, und die Extension wird nur durch ein Auseinandergehen mehrerer Röntgenmarker sichtbar. Ein weiterer Vorteil aktiv fixierbarer Elektroden ist die freie Wahl des Implantationsorts, während die Implantation passiv fixierbarer Elektroden auf RV-Apex und RAA beschränkt ist.

## Fazit

Die Wahl der Elektrodenposition im RV und RA erfordert eine umfassende Berücksichtigung der individuellen anatomischen Aspekte, der gewünschten Aktivierungssequenzen und Komplikationsrisiken.

Insbesondere im Kontext eines hohen antizipierten ventrikulären Stimulationsanteils oder bereits eingeschränkter LV-Funktion sollte die septale der apikalen Stimulation vorgezogen werden. Die Platzierung und die abschließende Beurteilung der Elektrodenposition sollten dabei in verschiedenen Projektionen erfolgen, um Fehlplatzierungen und somit potenzielle Komplikationen zu vermeiden. Das ausreichende Spiel der Elektroden spielt dabei sowohl für die akute als auch für die dauerhafte Stabilität eine entscheidende Rolle. Zusätzlich bieten intrakardiale Elektrogramme wertvolle Informationen, die neben den Standardmessungen für eine prognostisch zuverlässige Fixierung und Stimulation herangezogen werden können.
